# Role of the Internet in Solving the Last Mile Problem in Medicine

**DOI:** 10.2196/16385

**Published:** 2019-10-28

**Authors:** Bradford William Hesse

**Affiliations:** 1 National Cancer Institute Rockville, MD United States

**Keywords:** connected health, implementation science, patient engagement, community improvement, citizen science, digital divide, learning health care system

## Abstract

Internet-augmented medicine has a strong role to play in ensuring that all populations benefit equally from discoveries in the medical sciences. Yet, data from the Centers for Disease Control and Prevention collected from 1999 to 2014 suggested that during the first phase of internet diffusion, progress against mortality has stalled, and in some cases, receded in rural areas that are traditionally underserved by medical and broadband resources. This problem of failing to extend the benefits of extant medical knowledge equitably to all populations regardless of geography can be framed as the “last mile problem in health care.” In theory, the internet should help solve the last mile problem by making the best knowledge in the world available to everyone worldwide at a low cost and no delay. In practice, the antiquated supply chains of industrial age medicine have been slow to yield to the accelerative forces of evolving internet capacity. This failure is exacerbated by the expanding digital divide, preventing residents of isolated, geographically distant communities from taking full advantage of the digital health revolution. The result, according to the Federal Communications Commission’s (FCC’s) Connect2Health Task Force, is the unanticipated emergence of “double burden counties,” ie, counties for which the mortality burden is high while broadband access is low. The good news is that a convergence of trends in internet-enabled health care is putting medicine within striking distance of solving the last mile problem both in the United States and globally. Specific trends to monitor over the next 25 years include (1) using community-driven approaches to bridge the digital divide, (2) addressing structural disconnects in care through P4 Medicine, (3) meeting patients at “point-of-need,” (4) ensuring that no one is left behind through population management, and (5) self-correcting cybernetically through the learning health care system.

## Introduction

The internet has a strong role to play in ensuring that all populations benefit equally from discoveries in the biomedical sciences. To illustrate why this is the case, consider data from the US-based Centers for Disease Control and Prevention (CDC). In 2017, the CDC revealed that despite scientific progress on multiple fronts, certain portions of the population—especially those living in isolated, nonmetropolitan areas—were falling behind across seven of the leading causes of death in the country [1]. This backward regression in mortality outcomes is especially evident in the case of cancer. From 1980 to 2014, mortality data from the cancer registries revealed that while age-adjusted mortality rates were falling in urban and suburban areas (where access to medical services and communications infrastructure was prevalent), they were atrophying or even receding in rural areas of the country. New cancer hotspots began to emerge across the country nestled within the *hollers* of Appalachia, the *bayous* of the Mississippi Delta, and the vast geographic territories covered by Native American tribal lands [2,3]. The application of extant medical knowledge was impeded by the tyranny of distance. This is not just a US phenomenon. Meta-analyses from studies conducted worldwide have revealed that in the case of cancer, people living over 50 miles away from the nearest hospital tend to present with later stages of disease, experience unaddressed complications during treatment, experience lower quality of life, and fail to comply with prescribed pharmaceutical treatments [4].

Overcoming the limitations of an inadequate, industrial age supply chain for medical knowledge can be framed as the solving the “last mile problem” in health care. The good news, according to health services researcher Don Berwick, is that the rapid diffusion of internet technologies over the past two decades is beginning to put a solution to the last mile problem within our grasp. As he indicated in his testimony to the US President’s Cancer Panel, “There is now a worldwide collection of efforts, which is showing how much we can leverage knowledge through (health information technology) so that literally the best knowledge in the world can reach everyone in the world, at low cast, and at no delay.” [5]. This is the promise that those of us working at the intersection of medicine and the internet can help fulfil with vision and collaboration. The objective is worth our collective efforts. With respect to the evocative case of cancer, the American Cancer Society estimated that solving the access issue in oncology would improve mortality rates by approximately 22% per year [6].

## From Telemedicine to Connected Health: The Last 25 Years

In its earliest days, the idea of reaching remote populations through electronic means—a concept embodied in the practice of telemedicine—seemed to be a hopeful, but often an impracticable, solution for solving the last mile problem. Video-conferencing capabilities were expensive and technologically challenging, electronic health record (EHR) systems were rare, and patients’ abilities to reach their clinical teams through electronic communication were practically nonexistent. The world’s medical knowledge was still locked up in the stacks of academic medical libraries, which were often nonexistent in rural settings and inaccessible to practitioners in low-resourced countries. Patients were generally precluded from accessing medical knowledge directly, because they lacked access to professional distribution channels and the material was written in a highly specialized medical language that was inaccessible to anyone without a medical education. These industrial-age dissemination channels forced reliance on highly trained clinical personnel as mediators of medical knowledge and awarded a premium to the elite medical schools as purveyors of evidence.

In the early 1990s, the US-based National Science Foundation (NSF) invested in a strategy that would overcome the limitations of geography in science by connecting the world’s knowledge resources through a hyperlinked lattice of documents, data, remote devices, and personal communications. Under the leadership of Donald Lindberg, the National Library of Medicine joined the NSF in its vision for accessible online knowledge by digitizing its holdings and making them available in its online bibliographic resource, MEDLINE. The objective of MEDLINE was to help remote practitioners overcome the limitations of underresourced libraries and to help all communities benefit from an up-to-date, comprehensive snapshot of the extant medical knowledge base. Soon afterward, legislative and regulatory bodies made a set of decisions that would open the internet to the general public. Public-facing search engines directed anyone with a computer and a modem to the same bibliographic databases being used by their doctors. Biomedical visionaries predicted that electronic health would soon take its place along with electronic commerce as the new distribution channel for medical knowledge in health generally [7-9] and cancer specifically [10,11].

As the internet matured, it entered the mainstream of commercial, civic, and social life with substantial repercussions throughout. Initial forays into electronic commerce stumbled, bringing about the dot.com implosion at the beginning of the millennium, but as companies returned to the first principles, the practice flourished. In medicine, demand seemed to precede supply as patients flocked to the Web first, even before visiting their doctors, in search of reliable guidance on how to care for themselves or their families [12]. Patients’ online reconnaissance was not always appreciated by the medical establishment, which had been resisting the digitization of its own internal processes. In the absence of receptivity from the medical establishment, patients found each other online, exchanging insights through bulletin boards, chat rooms, and eventually social media. “Health 2.0” took on the form of a grassroots effort to encourage greater acceptance of engagement and innovation by patients across the supply chain [13]. In the United States, it would (literally) take an act of Congress to create the incentives for hospital systems and individual physicians to move from paper-based records to EHRs [14]. Even then, the early functionality of these EHRs would be oriented primarily toward billing and coding purposes. They were not designed with the appropriate human factors expertise to ease pressures on workflow [15] or to empower patients [16].

Now, 10 years after passage of the Health Information Technology for Economic and Clinical Health Act [17] in the United States, the conditions appear to be in place for substantial innovation to improve the medical knowledge supply chain. Adoption of EHRs in medicine has reached an all-time high, with 96% of nonfederal acute care hospitals and 86% of office-based physicians attesting to the meaningful use of health information technology [18]. Usability and safety issues are no longer swept under the table but have taken on a more centralized role in contemporary legislation [19]. Access to smartphone technology skyrocketed after the introduction of the iPhone in 2007, giving patients always-on, always-present access to the functionality of internet-based channels [20]. The wearable device and medical sensor markets have also been growing, giving medical entrepreneurs an opportunity to extend care more seamlessly into the home. The recent entry of a major device and software manufacturer into the personal health record market prompted the US General Accounting Office to declare that the market around consumer engagement in medicine may have finally reached a tipping point.

## Solving the Last Mile Problem: The Next 25 Years

Despite a tortuous path in bringing medicine into the dawn of the digital age, the conditions are now in place to make exponential progress in solving the last mile problem in health care. The following are some of the areas worthy of emphasis and continued monitoring over the next 25 years:

### Using Community-Driven Approaches to Bridge the Digital Divide

In the United States, the FCC’s Connect2Health Task Force has been monitoring the extent to which broadband capacity is available throughout the country to support the digitization of health care. Paradoxically, many of the same rural counties experiencing a decline in progress against the biggest threats to mortality according to the CDC are also falling outside the reach of reliable broadband coverage. These counties are experiencing a dual burden: The mortality burden from chronic disease is higher in these counties than in their metropolitan counterparts, while the infrastructure to reach these patients through community hospitals or, now telemedicine, is dwindling. In 2017, the FCC and the National Cancer Institute initiated a broadband pilot program in rural Appalachia called “Linking and Amplifying User-Centered Networks through Connected Health,” or LAUNCH. The program is using expertise from the Design Lab at the University of California San Diego; expertise for value-based partnerships from Amgen; oncology expertise from the Markey Cancer Center in Lexington, Kentucky; and channeled expertise from industry leaders to create a platform for community-driven development on top of an extended platform for connected services through broadband [21].

### Addressing Structural Disconnects in Care Through P4 Medicine

Twentieth century medicine bore the hallmarks of the industrial age, with one-size-fits-all solutions dominating the marketplace as blockbuster products and fee-for-service treatment centers offering the promise of indemnified cures, or repairs, after symptoms became too bothersome to ignore. The trouble was that these solutions, which were reactionary in nature and delivered too late in the disease process to prevent irreparable damage, were insufficient to cope with the deluge of noncommunicable diseases projected to drain the coffers of social support systems internationally. Leaders in medicine have called for a new approach, one enabled by the 21st century internet technologies. The new approach must be predictive, using cutting edge analytics to forecast risk; preemptive, utilizing prevention and early detection formulae to act upon those risk profiles before damage occurs; personalized, tailoring treatments to patients’ risk and preference profiles to create solutions that are both efficacious and value congruent; and participative, embracing the collaborative capacity of internet platforms to support patient engagement, community improvement, and citizen science [22]. To enable this new brand of medicine (referred to by some as “P4 Medicine”), health system engineers must focus on eliminating the disconnects in care that have otherwise derailed efficacious, empowered action across care teams inclusive of patients and their caregivers [23]. Just as FedEx and Amazon dominated the marketplace by ensuring custodial responsibility across the consumer fulfillment supply chain, new leaders in biomedicine will be those who eliminate the structural disconnects in care to ensure custodial support for covered lives across the interconnected supply chains delivering preemptive and participative care.

### Meeting Patients at the Point of Need

Just as internet-supported medicine can be mobilized to solve the last mile problem in terms of geography, data suggest that it can also be used to solve access issues as imposed by temporal constraints. Twentieth century medicine was dominated by the office visit or hospital stay, the clinical equivalent of bricks-and-mortar service delivery. The problem is that most patients’ health decisions occur outside of the clinical context [24]. NCI-funded clinical trials have already demonstrated just how effective the use of asynchronously collected patient-reported outcomes can be in helping cancer patients stave off the unanticipated side effects of treatment while reducing costs from preventable hospitalizations or controllable symptomologies. New efforts are underway within the biomedical technology sector to create the next generation of biologic sensors that can be used to place an electronic safety net around patients when they are away from the clinic. Usability engineers are designing more patient-centric ways for patients to ask questions and manage their care asynchronously while on travel or at home. Advanced medical sensors are being developed using nanotechnology (eg, microneedle sensors) to reduce the obtrusiveness of physiologic monitoring, while connected smart devices in the home are being engineered to detect deviations in air quality, falls, or complications from treatment passively and unobtrusively [25,26]. Tracking and understanding how these internet-connected devices can be used to support better patient outcomes through ongoing support outside of the home, while protecting privacy and safety, was a priority embedded within the 21st Century Cures Act passed in 2016 [27]. Further integrating the data from these devices into a supportive platform for personal care management and remote clinical monitoring without overwhelming the health care system will be the human factors challenge for the next 25 years.

### Ensuring That Nobody is Left Behind Through Population Management

When meaningful use requirements were first articulated for the incentives intended to spur adoption of Health Information Technology in the United States in 2009, one essential policy lever was included that pointed to a dramatic restructuring of the way medical care is delivered. The lever was population health management. Its purpose was to provide health insurers and health care providers the ability to go beyond individual patient management to a level of proactively managing the health and welfare of all patients within a specific practice, or to ensure that all patients are served equitably within the population of a health care plans’ members. Dr Nirav Shah, who was the Vice President for Kaiser Permanente in Southern California, delivered a poignant example of successful population health management in testimony to the President’s Cancer Panel in the spring of 2015. In his example, Kaiser used the tracking capacity of its mature EHR system to monitor patients’ recommended eligibility for age-/risk-based cancer screening. Office staff proactively followed prompts from the system to ensure that everyone on the list had been contacted with a recommendation for the screening. Results showed a 6-fold increase in mammogram testing, a 6-fold increase in cervical cancer testing, and a 10-fold increase in colorectal cancer testing. External reviews revealed an absence of disparate outcomes for the screening exercise; the approach benefitted all population with the membership equitably [5].

### Self-Correcting Cybernetically Through the Learning Health Care System

The other quantum leap forward enabled by connective technology is the premise that data volunteered by patients and harvested from administrative systems can be used to improve the performance of health care. Many of the largest consumer-facing electronics systems routinely request consent to gather data on reported system errors to improve the fidelity of their products in the field. In the era of internet-connected things, usage data from passive sensors can be used to adjust load levels at the community level, while data from personal voice assistants can be fed into machine-learning algorithms to improve sensitivity and performance of automated services. PatientsLikeMe.com, billed as the world’s largest personalized health network, helped spark a revolution in biomedicine by bringing citizen science to bear on the development and postmarket monitoring of life-saving therapeutics. The National Institutes of Health plunged into this revolution as it launched the All of Us initiative, a program designed to bring volunteered data from a million-patient cohort directly into the discovery engines of biomedicine. Taken together, these emerging capacities provide an early vision for how patients, providers, and scientists can participate together to realize what the National Academy of Medicine has referred to as a true Learning Healthcare System [28-30].

## Conclusion and Caveat

Data from the CDC offer a poignant reminder to the limits of industrial age medicine. Traditional supply chains are slow, expensive, and restrictive in their abilities to translate the benefits of hard-won medical knowledge equitably to all patients, regardless of where they live or what time constraints govern their days. As a result, broad swaths of the world’s population are being left behind, receiving neither the benefits of evidence-based knowledge nor the opportunity to participate equitably in the discovery of tomorrow’s cures. This is the last mile problem in medicine. A new paradigm, enabled by internet technology, brings hope that medicine can work collectively to solve the last mile problem over the next 25 years.

This hope, however, comes with a caveat. The same conditions that gave patients direct access to the scientific literature otherwise sequestered in the world’s most prestigious libraries, have also given rise to a social milieu in which medical misinformation can spread as quickly as medical fact [31]. Similarly, the same technology that allows for precision cataloging of a patient’s personal genome to be considered in tandem with biologic data from implanted sensors or contextual data from wearable device is rapidly creating an explosion of data, which, if left untethered, may prove to be paralyzing to decision makers [32]. Hard work will be needed to track the unanticipated consequences of exponential disruption in the medical space and then use the best principles of human-centered design to address them directly for the benefit of patient outcomes and public health. I am heartened to know that at that point, we will be able to read about the fruits of this labor in the publications of the Journal of Medical Internet Research.

## Abbreviations

CDC: Centers for Disease Control and Prevention
EHR: electronic health records
FCC: Federal Communications Commission’s
NSF: National Science Foundation
